# Membrane proteomic profiling to identify candidate therapy targets for glioblastoma infiltration

**DOI:** 10.1093/noajnl/vdag181

**Published:** 2026-07-10

**Authors:** Harry Porter, Kayley Mulhall, Maria Shah, Jeffy Joseph Vinohar, Konstantinos-Panagiotis Karadimas, Shaylen Mistry, Simon Deacon, Farhana Haque, Emyr Bakker, David J Boocock, Clare Coveney, Robert Layfield, Ruman Rahman, Phoebe McCrorie

**Affiliations:** Biodiscovery Institute, School of Medicine, University of Nottingham, Nottingham, UK; Biodiscovery Institute, School of Medicine, University of Nottingham, Nottingham, UK; Biodiscovery Institute, School of Medicine, University of Nottingham, Nottingham, UK; Biodiscovery Institute, School of Medicine, University of Nottingham, Nottingham, UK; Biodiscovery Institute, School of Medicine, University of Nottingham, Nottingham, UK; Biodiscovery Institute, School of Medicine, University of Nottingham, Nottingham, UK; Biodiscovery Institute, School of Medicine, University of Nottingham, Nottingham, UK; The Medical School, College of Health and Science, University of Lincoln, Lincoln, UK; The School of Medicine and Dentistry, University of Lancashire, Preston, UK; John van Geest Cancer Research Centre, The Centre for Systems Health and Integrated Metabolic Research (SHiMR), Nottingham Trent University, Clifton, Nottingham, UK; John van Geest Cancer Research Centre, The Centre for Systems Health and Integrated Metabolic Research (SHiMR), Nottingham Trent University, Clifton, Nottingham, UK; School of Life Sciences, Queen’s Medical Centre, University of Nottingham, Nottingham, UK; Biodiscovery Institute, School of Medicine, University of Nottingham, Nottingham, UK; Biodiscovery Institute, School of Medicine, University of Nottingham, Nottingham, UK

**Keywords:** glioblastoma, membrane, molecular docking, surface proteome, tumor infiltration

## Abstract

**Background:**

Glioblastoma (GBM) is an aggressive brain tumor characterized by rapid growth and infiltration. New therapies are desperately needed to improve GBM patient outcomes. Intra-tumoral heterogeneity is a common driver of failure for novel GBM treatments, and many preclinical studies rely on cell lines established from the tumor core. As a result, they fail to characterize the infiltrative tumor cells, which remain post-surgery and ultimately drive tumor recurrence.

**Methods:**

This study characterizes the membrane proteome of 3 patient-derived GBM cell lines isolated from the tumor invasive margin (GIN8, GIN28, and GIN31), which is a proxy for residual disease post-surgery. We combined plasma membrane protein analysis with total protein analysis using liquid chromatography-mass spectrometry to uncover therapeutic targets most amenable for drug repurposing. Molecular docking analysis predicted specific binding pockets on the surface of key membrane proteins against which the top 10 approved drug candidates were screened based on their binding energy scores.

**Results:**

Membrane proteins such as EDIL3, DYSF, ROBO1, SERPINE2, LOXL1, and CD70 were consistently significantly upregulated across GBM cell lines relative to healthy astrocyte controls, indicating potential functional roles in GBM progression. Molecular docking identified nilotinib (targeting LOXL1) and darifenacin (targeting SH3KBP1) as candidate drugs that can bind to identified membrane proteins. Nilotinib and darifenacin produced average IC_50_ values of 8.45 µM and 46.77 µM, respectively, in cell viability assays against GIN cell lines.

**Conclusions:**

These findings suggest that targeting membrane proteins offers promise for developing effective GBM therapies predicated on the most prognostically relevant intra-tumor region.

Key Points

- Multiple upregulated plasma membrane proteins identified in glioblastoma invasive margin cell lines.
- Drug docking analysis identified efficacious therapeutics against these proteins.
- Plasma membrane proteins thus represent directly amenable therapeutic targets.

Importance of the Study

Glioblastoma is an extremely heterogenous disease that is uniformly fatal. The GBM invasive margin has previously demonstrated similar characteristics to recurrent disease, which ultimately leads to patient fatality. Targeting cells within the invasive margin could increase time to recurrence, thereby increasing patient quality of life. Plasma membrane proteins on the cell surface offer a readily amenable drug-target, negating the need for therapeutics to enter the cell. Furthermore, proteins at low or non-existent levels on healthy brain cells, such as astrocytes, provide safer therapeutic targets by reducing off-target toxicity. This study develops and validates a pipeline by which a new avenue of efficacious therapeutics can be uncovered against glioblastoma invasive margin cell membrane proteins to be prioritized for preclinical to clinical translation.

GBM is a highly malignant form of primary brain tumor, with dismal median survival globally. GBM is classified as a Grade 4 astrocytoma by the World Health Organization^1^ indicating its high degree of malignancy and poor prognosis. Originating from astrocytes, GBMs are characterized by rapid diffuse growth and infiltration into adjacent brain parenchyma. This aggressive nature renders GBM particularly challenging to treat and manage. GBM cells are characterized by marked cellular diversity and genomic instability, which promotes both proliferative and infiltrative phenotypes.^2^

Enabling visualization and maximal safe tumor resection, 5-Aminolevulinic Acid (5-ALA) accumulates in malignant glioma cells and converts to fluorescent protoporphyrin IX. Tissue from the 5-ALA–defined invasive margin provides experimentally valuable material representing residual disease, offering a clinically relevant model for studying recurrence-driving tumor regions, as reported by us.^3-5^ As such, invasive margin cells represent a clinically relevant testbed for tumor regions that have historically remained refractory to treatment.

Current GBM treatment has not changed since the implementation of the Stupp protocol in 2005. This consists of maximal safe surgical resection, followed by oral temozolomide (TMZ) and concomitant radiotherapy. Despite a modest increase in survival time, TMZ efficacy is dependent on patients’ O6-methylguanine-DNA methyltransferase (MGMT) methylation status. As a result, survival times have remained at ∼16 months from diagnosis for almost 20 years, despite substantial efforts to better understand the tumor and identify new therapeutic options.^6^^,^^7^

GBM proteomics involves the comprehensive analysis of protein expression in GBM cells, utilizing advanced techniques such as mass spectrometry. This approach has identified critical biomarkers and dysregulated pathways, enhancing our understanding of GBM pathophysiology. Key studies have highlighted overexpression of EGFR and alterations in the PI3K/AKT/mTOR pathway.^8^ Additionally, proteomic profiling has revealed potential therapeutic targets and prognostic markers.^9^^,^^10^ Daubon et al identified 152 human proteins differentially expressed between core and invasive cells in a patient-derived xenograft mouse model highlighting the need to study intra-tumor heterogeneity in invasive GBM.^11^ However, this work, like many other GBM proteomics studies, relied on GBM samples derived from the proliferative core (which is surgically removed) and peripheral zone (non-5-ALA defined) but critically not the 5-ALA-defined invasive margin (proxy for residual disease).

Protein mass spectrometry is an important technique for fundamental and biomarker research in the study of GBM. Mass spectrometry has the potential to either supplement or eventually replace conventional approaches for determining the presence, stage, and subtype of GBMs. Its high sensitivity and capacity to identify multiple protein biomarkers supports this notion.^12^ Ghosh et al presented a molecular signature for GBM based on transmembrane proteins localized within the plasma membrane from patient tumor ­samples. They reported a 33-protein signature using REMBRANDT (*n *= 228) and The Cancer Genome Atlas (TCGA) patient datasets (*n *= 547), which differentiated GBM samples from control samples, whereby 4 plasma membrane proteins (CD44, VCAM1, HMOX1, and BIGH3) were differentially detected in the blood of patients pre- and post-tumor resection, demonstrating potential as clinical biomarkers and also indicating the feasibility of LC-MS for biomarker detection in liquid biopsies.^13^

Here, we assessed label-free, data-independent acquisition (SWATH) mass spectrometry to elucidate protein expression of GBM infiltrative cells. To ensure direct therapeutic amenability, we conducted proteomic profiling of both total protein and membrane protein fractions, whereby plasma membrane proteins were resolved from the broader membrane fraction in downstream data analysis steps; these plasma membrane proteins represent proteins most visible to drug targeting. To ensure future candidate repurposed drugs are safe, we prioritize surface proteins that are not detected in non-disease cells (control human astrocytes [HA]) or only expressed at significantly lower levels than infiltrative GBM cells, thus representing viable therapeutic windows. Therefore, our overarching hypothesis states that membrane proteomics will reveal new GBM therapy targets amenable for rapid drug repurposing and predicated on the infiltrative tumor margin. Molecular docking techniques are used to determine repurposed therapeutic candidates against the identified GBM therapy targets, and potency assessed with cell viability assays.

## Methods

### *GBM* In Vitro *Culture*

Culture conditions were maintained at 37°C with 5% CO2 and 90% relative humidity. **G**lioma **IN**vasive margin cell lines (GIN8, GIN28, and GIN31) were isolated from the 5-ALA positive invasive margin, where fluorescent cells can be seen to infiltrate surrounding healthy brain tissue, from 3 different patients (see Supplementary Table S1) H&E stained tissues are displayed in Supplementary Figure S1, demonstrating tumor locations from which the GIN lines are derived from. Cell lines were cultured in Dulbecco’s Modified Eagle Medium (DMEM; Sigma-Aldrich, St. Louis, MO, USA) supplemented with 10% FBS, 1 g/L glucose, and 2 mM L-glutamine (Sigma-Aldrich). Cells were used within a passage window of 5. Although cell pellets were collected at the same time, the cell lines were not compared at the same passage. Cell lines were derived at the University of Nottingham and were comparable to their respective primary tissue on STR.^4^

### *HA* In Vitro *Culture*

Primary human cortical astrocytes (HA, Sciencell, SC-1800) were grown as per manufacturer’s instructions. Briefly, HA were grown in Poly-L Lysine coated cell culture flasks in astrocyte medium containing 2% FBS and 1x astrocyte growth supplement at 37°C with 5% CO_2_. Cells were used within a passage window of 5.

### Extraction of Total Cellular and Total Membrane Proteins

For each biological repeat cells were pooled from 3× 70% ≥ confluent T-175 flasks and stored at −80°C. Cell pellets were washed once with 3 mL of cold PBS and resuspended in 2 mL of Abcam Homogenize Buffer Mix (ab65400) supplemented with protease and phosphatase inhibitors and resuspended. Cells were centrifuged at 700 × g for 10 minutes at 4°C. The formed pellet represented total protein. Cell supernatant was extracted and centrifuged for 10000 × g for 30 minutes at 4°C. The formed pellet represented the total membrane protein fraction, which was resuspended in 60μL of 0.5% Triton X-100 in PBS and then stored at −80°C.

### Protein Quantification Using BCA Assay

Total and membrane protein fractions were mixed with 60-100 µL of the Abcam Homogenize Buffer mix (ab65400) supplemented with protease and phosphatase inhibitors, incubated on ice for 15 minutes, and sonicated for 10 seconds. The lysates were centrifuged at 16100 × g for 20 minutes, and the supernatant was collected for protein concentration determination.

Total protein and membrane protein concentrations were measured using the bicinchoninic acid (BCA) assay. Bovine serum albumin (BSA) standards were prepared, ranging from 0 to 2 mg/mL. Samples were diluted 1:10 with distilled water, and 10 µL of each standard and sample were added in triplicate to a 96-well plate. BCA reagents were added according to the manufacturer’s instructions, and the plate was incubated at 37°C for 30 minutes. Absorbance was measured at 562 nm using a BMG LabTech Plate Reader with SpectroStar Nano Software and protein concentrations were calculated against a standard curve.

### LC-MS/MS-Based Proteomics

50 μg of cell lysate from total and membrane protein was used for LC-MS/MS identification of specific protein targets. Samples were processed through S-trap microcolumns (Protifi) according to manufacturer instructions, which include reduction, alkylation, and trypsin digestion on-column. Eluted peptides were vacuum-concentrated to dryness and reconstituted in 200 uL at a final concentration of 5% acetonitrile: 0.1% formic acid (2 μL, ∼500 ng protein digest/peptides) were directly injected by autosampler (Waters M-class) and gradient elution at 10 μL/min (Phenomenex Kinetex XB-C18 2.6 μm 15 × 0.3 mm analytical column, 30°C, mobile phase A 0.1% formic acid: mobile phase B acetonitrile with 0.1% formic acid LCMS grade) onto a Sciex ZenoTOF 7600. Gradient elution with the following profile: 3% B to 35% over 12 min, 80% B at 13-15 min, and re-equilibration at 12 μL/min to the starting conditions at 15.5 min for a 16.5 min total runtime. MS analysis was via the Optiflow source with a microflow 1-50 μL electrode in positive SWATH mode with zeno pulsing enabled at 4700 V, using 65 variable SWATH windows optimized on a complex human protein lysate from m/z 400-750 12 ms per window following a TOFMS scan of 25ms for a total cycle time of 1.146 s. Ionization was deactivated at 14.2 min to reduce contamination during the column wash.

### Proteomic Data Analyses

MS acquisition files were processed in DIA-NN 1.9 to generate Max LFQ intensities for each protein. Differentially expressed proteins between GIN cell lines versus HA were identified using Amica (version 3.0.1) using the limma package, with adjusted *P*-value <.05 and log2 fold-change (log2FC) >2 thresholds, and commonly identified contaminants in proteomics experiments were excluded from further analysis based on cRAP database IDs. Multiple hypothesis testing was adjusted for using the Benjamini & Hochberg method, number of biological replicates, and significant differentially abundant proteins with and without BH correction of *P*-value (Supplementary Table S2). Missing values were addressed by requiring at least three valid values in one group, followed by normal imputation. This method introduces random noise (downshift 1.8, width 0.3) to reflect uncertainty while preserving variability. Positive fold changes denote higher expression in malignant cells, and conversely reduced expression in HA. Heatmaps of differentially expressed proteins were plotted using the R package pheatmap (version 1.0.12) and ComplexHeatmap (version 2.22.0).

Principal component analysis (PCA) and data processing/visualization was conducted in R (version 4.4.1). Graphs were produced using the ggplot2 package (version 3.5.1). PCA was conducted using the prcomp() function of the stats package (version 4.4.1), on log2 transformed protein abundance data from membrane and total protein samples separately. Intersection of protein lists was conducted in R using the venn() function of gplots (v 3.1.3.1) and the Reduce(intersect) function (base R). Venn diagrams were reproduced graphically in PowerPoint.

Identified proteins in the membrane protein fraction were classified as being plasma membrane proteins if annotated in Gene Ontology cellular compartment terms associated with the cell surface (GO: 0005886—plasma membrane, GO: 0009986—cell surface, GO: 0043235—receptor complex, and GO: 0055085—transmembrane transport). Pathway enrichment analysis comparing total and membrane protein fractions from the same cell line was conducted using fgsea (version 1.32.4) where proteins were ranked by log2FC from Amica DEA using C5 GO cellular compartment gene sets retrieved from MSigDB.

Pathway enrichment analysis of proteins expressed at a higher level in GIN cells compared to HA was conducted in clusterProfiler (version 4.14.6) with significantly upregulated defined as those with adjusted *P*-value <.05 and log2FC > 2 and universe specified as all proteins detected in either cell line in a given protein fraction.

### Analysis of Publicly Available GBM Clinical Datasets

Publicly available and clinically annotated GBM proteomics data from the Clinical Proteomic Tumor Analysis Consortium^14^ (Glioblastoma [CPTAC, Cell 2021]); and transcriptomics data (Glioblastoma Multiforme (TCGA, GDC) datasets were retrieved from the cBioPortal online platform^15^ (https://www.cbioportal.org/). Data was then analyzed in GraphPad PRISM 10.3.1. Log2 transformed, normalized, and batch-corrected proteomics data from the CPTAC GBM Discovery Study—Proteome including 10 unmatched normal brain samples was retrieved from supplementary data of.^16^

Published RNA-sequencing data composed of spatially resolved intra-tumor regions from 10 GBM patients, including the patient samples that generated the cell lines GIN28 and GIN31, has been previously published and was processed to a gene count matrix as described previously^5^ Counts were VST normalized using DESeq2 (1.48.1) and plotted using ComplexHeatmap (2.24.1) in R. Survival analysis results were obtained from Glioma-BioDP, based on the publication by Xiang Deng et al^17^^,^^18^ Software: Glioma-BioDP (https://glioma-biodp.nci.nih.gov). RNA sequencing data was retrieved from the IVY-GAP project (https://glioblastoma.alleninstitute.org/static/home), normalized gene-level Fragments Per Kilobase of transcript per Million mapped reads (FPKM) values were filtered in R to only include samples from the cellular tumor, infiltrating tumor, or leading edge and genes encoding the 18 common upregulated membrane proteins in GIN cells compared to control HA.

A list of proteins reported as being more abundant in primary GBM relative to normal adjacent tissue (NAT) was retrieved from the supplementary data of this publication, filtered consistently with the author’s original analysis (Thresholds: *Q* *<*  0.1, log_2_FC > 1) and the intersection with our 18 common membrane proteins compared using the R package VennDiagram (1.7.3).

Pathology data was retrieved from the Human Protein Atlas and filtered against proteins of interest in R. The pathology dataset (https://www.proteinatlas.org/download/pathology.tsv.zip) was filtered to include glioma samples only and ordered by the variable “prognostic—unfavorable” or “prognostic favorable.”

### Molecular Docking Analysis

The 3D crystal structure for each protein (attained by X-ray diffraction data on RCSB Protein Data Bank or predicted structures from AlphaFold) was first screened in DrugRep to identify specific binding pocket coordinates and subsequently screened against a library of approved drugs, searching for the top 10 drugs that were predicted to successfully bind to the desired binding pocket. PyRx (Version 1.0, ran on Windows 10) was employed to conduct targeted docking of approved drugs against these binding pockets. Successful drugs were filtered according to their binding energy score and rotatable bonds for PyRx docking. Successful candidates were then run through PyRx for multiple ligand docking to determine binding affinity and root mean square deviation (RMSD) values for the best confirmation. Drugs that passed this process were further narrowed down according to the following criteria in Table 1.

**Table 1.** Criteria used to refine candidate drugs obtained from PyRx docking for *in vitro* testing

Criteria	
**Pre-PyRx screening**	
Binding energy score (kcal/mol)	≤−7
Rotatable bonds	5-10
**Post-PyRx screening**	
Upper and lower RMSD	0
Binding affinity	≤−7
Previous evidence of cytotoxicity	Yes
Previous use in clinical trials	No
Hydrogen bond donors	≤3
Hydrogen bond acceptors	≤7
LogP	1.0-4.6
Molecular weight (Da)	150-550

To validate the molecular docking results, lurasidone (HY-B0032A-10mg) and darifenacin (T62320-10 mg) were purchased from Cambridge BioScience, whilst nilotinib (SML3799) was purchased from Merck.

### Western Blotting

CD324 (E-Cadherin, # 4-3249-82), A2M (**#** MA5-38211) LOXL1 (**#** LOXL-101AP) SH3KBP1 (**#** MA5-61977) were ordered from ThermoFisher Scientific. Samples were resolved on a Bio-Rad 8%-16% Mini-PROTEAN TGX Precast Protein Gel using 1× Tris/Glycine/SDS Running Buffer. A total volume of 20 µL of protein sample (∼7 ug total protein) was loaded per lane, and SeeBlue Plus2 Pre-Stained Protein Standard was utilized as the molecular ladder.

Proteins were transferred onto a nitrocellulose membrane via wet transfer using a Tris-glycine transfer buffer at a constant voltage of 30 V overnight (∼16 h) at 4°C. Following transfer, membranes were blocked for 40 minutes at room temperature in Thermo Scientific StartingBlock (PBS) Blocking Buffer.

Primary antibody incubation was performed for 75 minutes at room temperature. Following primary incubation, membranes were washed three times for 5 minutes each in Tris-buffered saline with 0.1% Tween-20 (TBST).

Membranes were then incubated in IRDye 680RD and 800CW secondary antibodies (1:10,000 dilution) for 1h at room temperature in the dark, followed by three final 5-minute washes in TBST. Blots were imaged using an LI-COR Odyssey Fc Imaging System. Whole-lane protein loading was verified using Ponceau S Stain and analyzed via ImageJ (Fiji).

### Cell Viability Assays

GIN8, GIN28, and GIN31 cells were seeded at 5000 cells per well in a 96-well plate and incubated overnight at 37°C with 5% CO_2_. The existing media was aspirated and replaced with 100 µL of drug-spiked media (1-200 µM) and left for 72 hours. Media was removed, and 100 µL of working concentration of Presto Blue Cell Viability Reagent was added to each well and incubated in the dark for 45 minutes at 37°C with 5% CO_2_. Fluorescence was measured **λ_ex_** 544 nm and λ**_em_** 590 nm using an Infinite 200PRO plate reader using Tecan i-control software. Cell viability was compared to a vehicle-only control.

## Results and Discussion

### Isolation of Membrane Proteins

**Figure 1.**
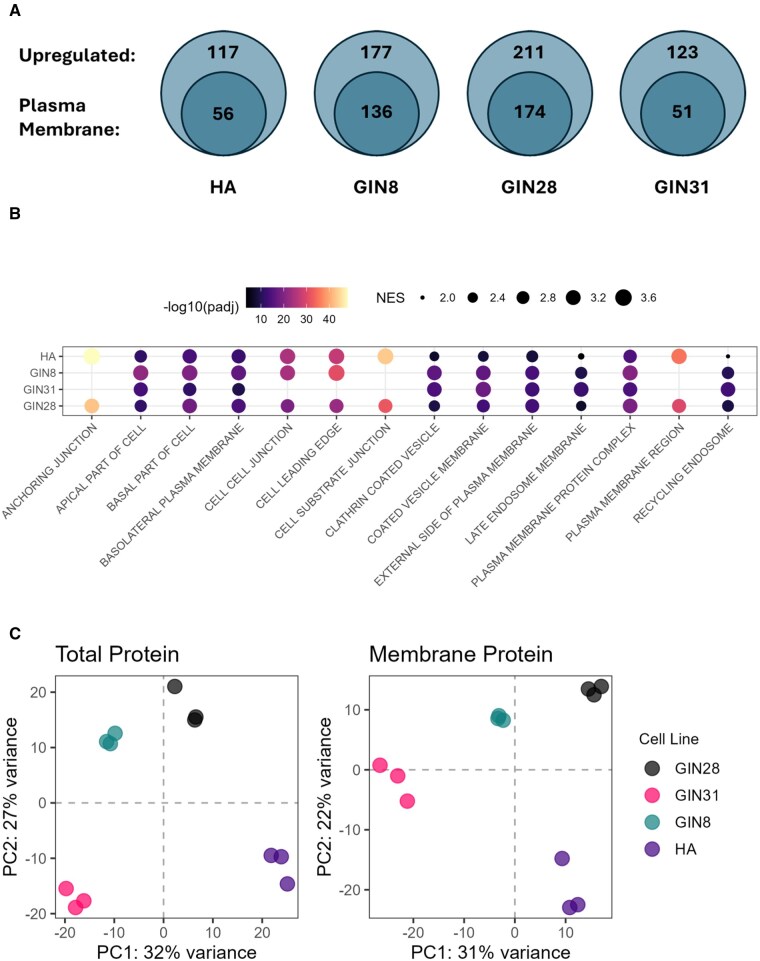
Membrane proteins were successfully extracted from control astrocytes and GIN cell lines. (A) Summary of proteins identified in the membrane and total protein fractions from HA, GIN8, GIN28 and GIN31 cell pellets. Inner circle indicates the number of significantly upregulated proteins (log2FC > 2, *P*-adj <.05) in the membrane protein fraction of each cell line relative to the total protein fraction which are validates as plasma membrane proteins based on gene ontology (GO) annotations. Outer circle indicates the number of other significantly upregulated proteins not associated with plasma membrane GO annotations. (B) Dotplot displaying GSEA comparing MP to TP for each cell line against cellular compartment GO terms. Only significantly enriched pathways (*P*-adj <.05, NES > 1) are shown. (C) PCA plots to show differences in total proteins and membrane proteins extracted from each cell line.

To comprehensively characterize the proteome of cells derived from the GBM invasive margin versus non-disease cells (HA), total and membrane protein fractions were isolated and analyzed. This facilitated enhanced recovery of membrane proteins while retaining the total protein as a reference representing proteins involved in signaling downstream of key membrane proteins of interest. We first set out to validate our membrane extraction methodology; by comparing relative abundance of proteins in the membrane and total protein fraction of each cell line (Figure 1A). This identified 173 significantly upregulated proteins in the membrane fraction derived from HA compared to total protein. Notably, of these, 32% could be mapped to gene ontology (GO) terms associated with the cell surface, indicating that these are plasma membrane proteins. Similarly, we observed upregulation of proteins associated with the plasma membrane in the membrane fraction of GBM cell lines of which 43%, 45%, and 29% were annotated as plasma membrane proteins for GIN8, GIN28, and GIN31, respectively.

Enrichment of plasma membrane proteins in the membrane protein fraction was further demonstrated by Gene Set Enrichment Analysis (GSEA) comparing each cell’s membrane protein sample to its respective total protein sample (Figure 1B). This revealed positive enrichment of proteins associated with GO cellular compartments relevant to the cell surface such as apical and basal parts of the cell, cell-cell junctions, and plasma membrane protein complexes. However, this also identified positive enrichment in terms associated with extracellular vesicles, highlighting that the membrane protein extraction methodology used in this work does not only enrich for plasma membrane proteins but other cell membranes. Therefore, while downstream analysis was conducted, including all membrane (including those found in the Golgi apparatus) proteins, plasma membrane proteins were prioritized based on increased ability for drug delivery.

Finally, PCA showed that each cell line clustered separately in both the total and membrane protein fractions (Figure 1C). We observed a similar clustering pattern in PCA plots between the total and membrane protein fractions, indicating that both protein fractions can be used to distinguish distinct cell types. Furthermore, both total and membrane protein fractions demonstrated significant variation between all 3 GIN lines, displaying inter-patient heterogeneity, which is a common facet of GBM.^19^ While PCA plots indicate distinct differences between the proteomes of GIN cell lines, the identification of ubiquitously upregulated proteins in GIN cells relative to HA offers a potential for druggable targets against infiltrative GBM cells, which are spared following surgical resection.

### Characterization of Membrane Proteins Specific to GIN Cells

**Figure 2.**
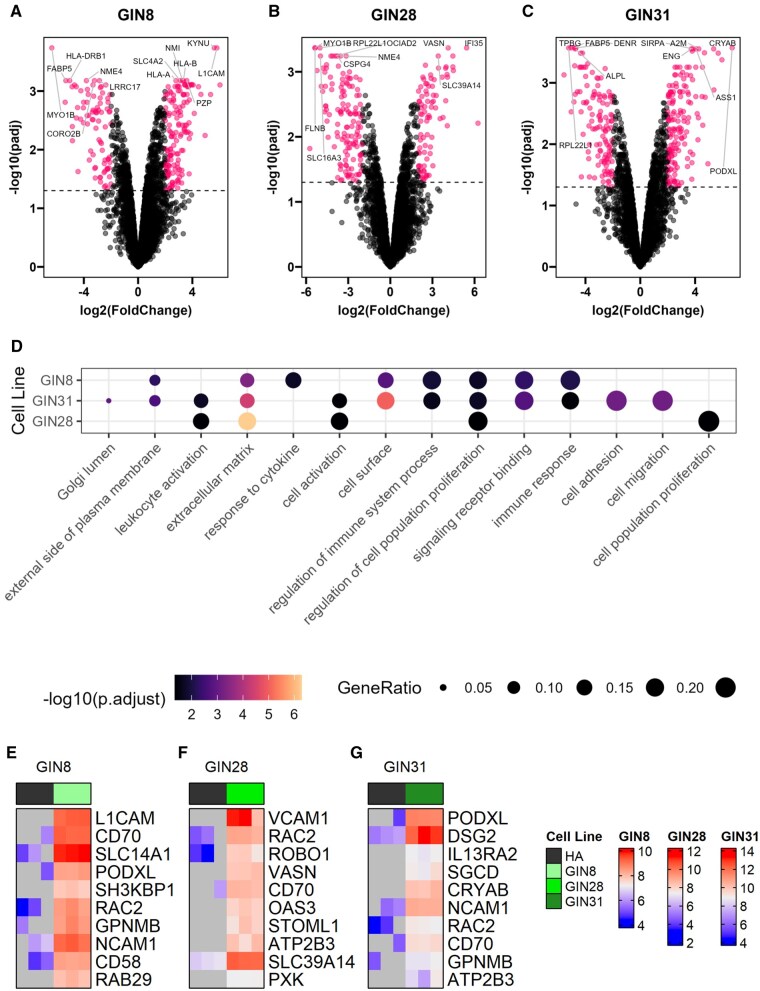
The membrane proteomic landscape shows significant differences in membrane proteins in GIN lines when compared to HA. Volcano plots showing differentially expressed membrane proteins in (A) GIN8 (145 Upregulated and 84 downregulated—of which 68 and 35 are known plasma membrane proteins) (B) GIN28 (82 upregulated and 130 downregulated—of which 28 and 66 are known membrane proteins) and (C) GIN31 (147 upregulated and 120 downregulated—of which 81, and 50 are known membrane proteins). Significantly changed proteins are shown in pink (absolute log2 fold change >2, *P-*adj value <.05) the top 5 (largest log2 FC) up and down regulated membrane proteins are annotated. (D) Dot plot showing enrichment analysis of significantly upregulated proteins (log2FC > 2, *P*-adj <.05) in the membrane fraction of each GIN cell line compared to HA. Enrichment analysis conducted on GO terms using clusterProfiler. Heatmaps showing the top 10 (highest log2FC) upregulated plasma membrane proteins in (E) GIN8, (F) GIN28, and (G) GIN31 cells compared to HA. Heatmap color indicates log2 transformed protein expression. Grey indicates that protein was not detected in this sample.

We next investigated differences in membrane protein expression between GIN cell lines and HA. As displayed in Figure 2A-C, differential abundance analysis identified significant differences in membrane protein expression, with 145, 82, and 147 significantly upregulated proteins in the membrane protein samples comparing GIN8, GIN28, and GIN31 individually to HA.

For GIN8, NMI, HLA-B, L1CAM, KYNU, and PZP were all significantly upregulated. High expression of NMI has previously been correlated with poor prognosis in GBM, whereby its expression was also correlated with EGFR amplification.^20^ Furthermore, L1CAM has also shown a significant correlation with poor prognosis in gliomas, whereby a putative role in chemoresistance, proliferation, and cell migration has been observed.^21^ GIN28 showed VASN, IFI35, ROBO1, ASPSCR1, and SLC39A14 were all significantly upregulated (top 5 ranked by *p*-adj value). These proteins have been reported to be associated with cell migration, immune and TGF-ß signaling, and cellular transport.^22^^,^^23^ Finally, GIN31 cells revealed ASS1, A2M, ENG, CRYAB, and PODXL as significantly upregulated when compared to HA. Roles of these proteins include amino acid biosynthesis, protease inhibition, and angiogenesis.^22^^,^^23^ Once again, this emphasizes the inter-patient heterogeneity observed in GBM patients.

In order to understand the functional relevance of upregulated membrane proteins in GIN cell lines compared to HA, pathway enrichment analysis was conducted on all upregulated proteins in the membrane fraction (Figure 2D), with corresponding upregulated proteins displayed in heatmaps in Figure 2E-G. GIN8 was defined by increased expression of membrane proteins associated with immune response and cytokine signaling, whereas GIN28 proteins were associated with Cell Population Proliferation and those in GIN31 were associated with cell migration. Notably, genes associated with regulation of proliferation and extracellular matrix were enriched in membrane proteins upregulated in all three GIN cell lines compared to HA. Furthermore, pathways for cell division, cell migration, and cell adhesion are all enriched, highlighting the invasive and proliferative phenotype of these cells left behind after surgery, which ultimately cause tumor reoccurrence and fatality.^24^ Interestingly, many immune response pathways are enriched; although previous work has shown that GBM cells can avoid the immune system,^25^ there is an absence of intentional immune stimulation in this *in vitro* setup, indicating a pleiotropic function to these proteins within these pathways.

### Investigation of Common Upregulated Proteins from GIN Cells

**Figure 3.**
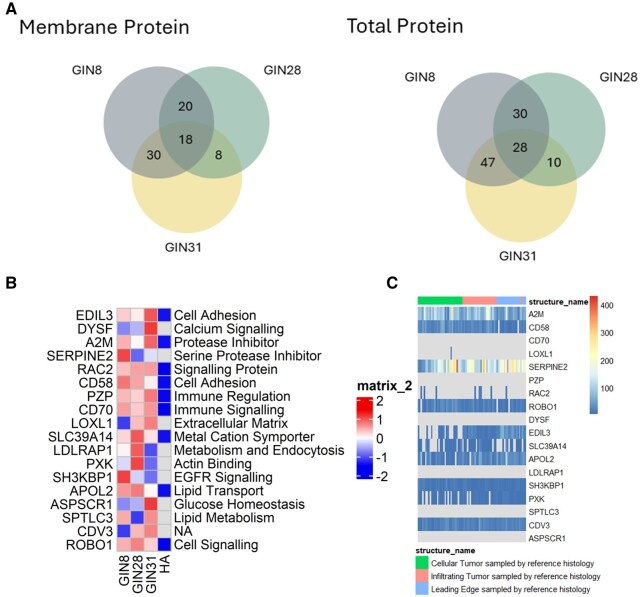
Common differentially expressed proteins are observed across all 3 GIN lines. (A) Venn diagrams showing common upregulated proteins between GIN8, GIN28 and GIN31 cells in membrane and total protein fractions. Based on the individual cell line analysis and thresholds in Figure 2A-C. (B) Heatmap showing mean expression of the 18 commonly upregulated membrane proteins in each cell line. Heatmap color indicates z-score normalized protein expression. Grey values indicate that protein was not detected in this sample. Functions retrieved from Human Protein Atlas. (C) Heatmap showing gene expression of the 18 commonly upregulated membrane proteins in tumor samples from the IVY Glioblastoma Atlas Project (GAP) database. Heatmap shows normalized fpkm values. Grey indicates genes not expressed in the dataset (fpkm less than 5).

Common upregulated plasma membrane proteins across all 3 GIN lines would provide evidence of key druggable targets on cells derived from the invasive margin from multiple patients. The three cell lines are derived from patients with differing survival outcomes, providing a small but representative cohort that reflects varied clinical responses. Similarities exist between patients, in that all have unmethylated MGMT status and intact ATRX. Figure 3A. shows Venn diagrams for common upregulated membrane and total proteins between the GIN lines. The analysis revealed a total of 18 membrane proteins in the dataset (13 of which were plasma membrane proteins, identified using Human Protein Atlas); their names and functions are displayed in Figure 3B.

Of the 18 membrane proteins detected across all 3 GIN lines, 9 proteins were not detectable in the HA cell population via LC-MS, as denoted by the grey boxes in Figure 3B: DYSF, SERPINE2, LOXL1, LDLRAP1, PXK, SH3KBP1, ASPSCR1, CDV3, and SPTLC3, which makes these proteins ideal candidates for therapeutic targeting.

Differential abundance analysis was also conducted on the total protein fraction identifying 232, 115, and 205 proteins expressed at a higher level in GIN8, GIN28, and GIN31 relative to astrocytes (Supplementary Figure S2A-D). Furthermore, 28 proteins in the total protein fraction were found to be upregulated in all three cell lines relative to astrocytes (Supplementary Table S3; Supplementary Figure S3). Notably, this included several known plasma membrane proteins, including PXK, RAC2, and CD70 which were also identified in the membrane protein fraction. Many of the proteins have known functions in cell division. For example, BRD2, which regulates proliferation and cell differentiation, has also been studied as a potential drug target in GBM. When inhibited with a small molecule compound, a significant change in proliferation and migration was observed in U87 cells.^26^ Furthermore, KIF20A, which has known roles in cytokinesis and intracellular transport, is commonly upregulated in gliomas, which has been shown to promote cell viability and invasion with potential chemotherapy-resistance effects.^27^

However, the focus of this study is toward the more readily targetable plasma membrane proteins found on the surface of GBM cells. Many of the 13 commonly upregulated plasma membrane proteins have roles within signaling pathways for metabolism and transport. Interestingly, SH3KBP1 has roles in EGFR signaling, which has previously been demonstrated as enhanced in GBM and many other cancers.^28^^,^^29^ Song et al reported that SH3KBP1 expression is associated with worse survival in GBM patients and is also prominently expressed in GBM stem cells. Silencing SH3KBP1 reduced GBM cell proliferation and migration *in vitro*, as well as reducing growth of xenograft tumors *in vivo*.^29^ Furthermore, previous work has shown that SH3KBP1 has roles in the invadopodium, which facilitates the invasion process of tumors,^30^ making it an attractive protein to target.

One candidate plasma membrane protein, ROBO1, has roles in axonal guidance and is associated with migration and invasion of GBM cells. It is upregulated in gliomas and has a negative correlation with survival time.^31^ CAR-T therapy against ROBO1 in recurrent GBM models has shown a doubling of median survival when compared to controls in cell-line derived xenograft models, indicating the role of ROBO1 in GBM proliferation. In the same study, ROBO1 expression was greater in the peripheral regions of the tumor, consistent with our findings in GBM cells derived from the invasive margin. Furthermore, a pan-PTP4A inhibitor (JMS-053) which targets the PTP4A-ROBO1 signaling axis, conferred inhibition of GBM cell growth *in vitro*, yet sparing neuronal stem cells. However, this inhibition did not translate *in vivo* due to poor penetration of the blood brain barrier, indicating that JMS-053 could be a good candidate for local drug delivery methods.^32-35^

### Upregulated Membrane Protein Expression in GBM Tissues

To address the limitations of using primary human fetal astrocytes and tumor cell lines serially passaged in FBS as models of non-disease brain and infiltrative GBM tissue, we investigated expression of the 18 common membrane proteins in published tissue datasets. Firstly, we confirmed mRNA expression of genes coding for 16/18 membrane proteins in 10 GBM patients for which, we previously conducted RNA-seq of distinct tumor regions^5^ (Supplementary Figure S4A). Notably, this included tissue from the tumor core and invasive margin in patients from whom GIN28 and GIN31 were derived.

While A2M and LOXL1 were not detected in our patient tissue dataset, A2M was shown to be expressed in GBM tissue from the Ivy Glioblastoma Atlas Project (Ivy GAP), including regions from the “infiltrating tumor” region (Figure 3C).^36^ The cells from the “infiltrating tumor” of the IVY GAP spatially RNA-sequenced tumors align closely with our cell lines derived from the invasive margin, however, samples from the tumor leading edge and core are retained for comparison. To this end, 11 out of the 18 commonly upregulated membrane proteins in our cell lines were present within the Ivy GAP cohort. Of these, SERPINE2 and PXK, which were not detectable in HA, showed a high presence in the cells of the infiltrating tumor. Finally, when inputting the 9 membrane proteins ubiquitously expressed in GIN cells, but not in HA, into the GBM Bio Discovery Portal, we found that CDV3, LDLRAP1, DYSF, and LOXL1 displayed a higher expression in the mesenchymal GBM subtype (data not shown). This is in concordance with previous data, which indicated that cells in the invasive margin align with the mesenchymal subtype.^5^

Assessment of membrane protein expressions using RNA-seq data is problematic, therefore, we additionally validated our 18 proteins against publicly available GBM proteomics datasets. Firstly, we were able to confirm expression of 16/18 membrane proteins in a cohort of 99 GBM and 10 healthy brain samples^16^; CD70 and SPTLC3 were not detected (Supplementary Figure S4B and C). Notably, hierarchical clustering based on these proteins was sufficient to separate normal tissue from most GBM tissue samples. Furthermore, we found that 3/18 membrane proteins (RAC2, DYSF, and ROBO1) were reported to be expressed at a higher level in GBM tissue relative to NAT, making them robust candidate targets shared across tissue samples and our invasive margin cell lines. While this did not validate CD70 and SPTLC3 expression in GBM proteomes, CD70 is an established GBM cell surface marker.^34^ To our knowledge, there are no available datasets from which membrane proteins are purified from GBM and “healthy” brain tissue that could be used as validation for our analysis. Critically, membrane proteins expressed at relatively low levels may not be detected for differential expression in conventional proteomics workflows.^37^ Therefore, future studies applying membrane proteomics on infiltrative GBM and non-tumor brain tissue sections would be beneficial to further advance this work. However, the consistency between our samples and existing GBM-associated membrane proteins provides increased confidence that the control chosen is adequate. Furthermore, investigating the significantly upregulated proteins that have not been linked with GBM previously could be of high relevance as we are specifically investigating cells derived from the GBM invasive margin, which has not been reported previously.

**Figure 4.**
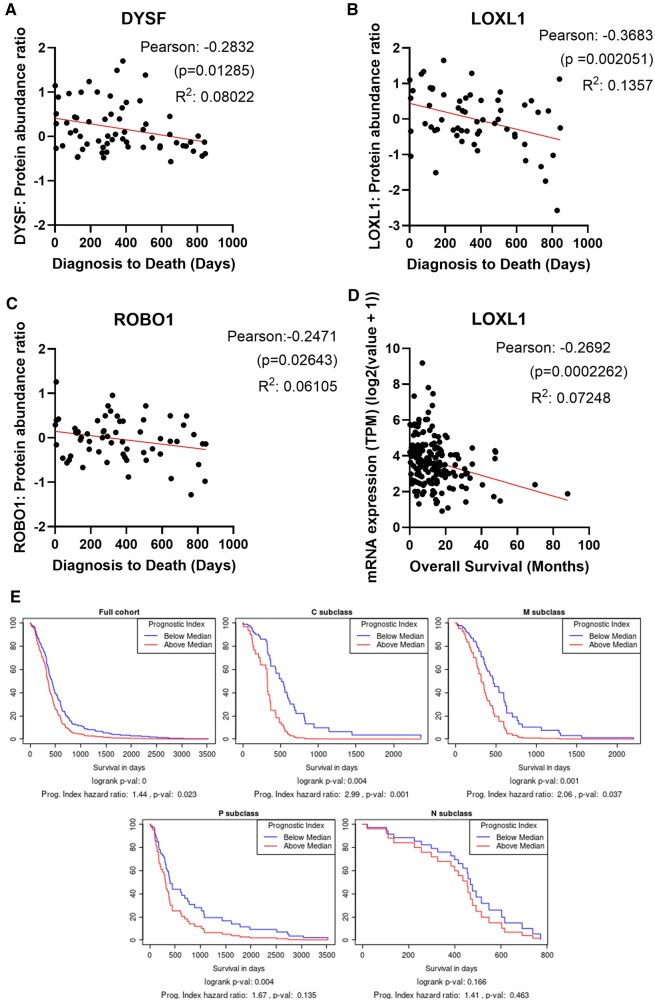
Investigation of translational potential of 18 membrane proteins upregulated in all three GIN cell lines relative to HA controls. (A-C) Analysis of correlation between protein abundance ratio and days from patient diagnosis to death in GBM clinical samples retrieved from the CPTAC study on cBioPortal. (A) DYSF: *n *= 62, (B) LOXL1: *n *= 59, (c) ROBO1: *n *= 62. (D) Analysis of correlation between LOXL1 mRNA expression transcripts per million (TPM) and Overall survival (months) in GBM clinical samples from TCGA-GBM cohort accessed through cBioPortal. *n *= 166. Pearson’s Rho, and *P*-value shown, linear regression was then applied to generate the trend-line shown in red (R^2^ value shown). (E) Survival analysis based on the impact of the multi-gene prognostic index when inputting the 18 upregulated membrane proteins into the National Cancer Institute Glioblastoma Bio Discovery Portal. Kaplan-Meier survival graphs stratified by >median or <median. Data separated from full cohort (logrank *P*-value <.001), to the four GBM subclasses: classical (‘C’) (logrank *P*-value .004), mesenchymal (‘M’) (logrank *P*-value .001), proneural (‘P’) (logrank *P*-value .004) and neural (‘N’) (non-significant; logrank *P*-value .166). No significance in each cohort’s MGMT hazard ratio was found.

Additionally, we aimed to identify if the membrane proteins expressed at higher levels in GIN cell lines have been identified as prognostic indicators. The 18 membrane proteins described in Figure 3 were screened against pathology data from the human protein atlas to identify whether they have prognostic value. Of the 18 proteins, only LOXL1 was found to be prognostic, with high LOXL1 mRNA expression associated with a worse prognosis (Kaplan-Meier analysis of correlation between mRNA expression level and patient survival [log-rank *P *= 9.519e-05], data not shown). However, we were interested in identifying whether expression of these proteins is important for GBM clinical outcomes at the protein level, rather than mRNA. We identified that proteomic data from 99 treatment naive GBMs^14^ was available for analysis in the cBioPortal platform (CPTAC, Cell 2021) dataset. All proteins, except CD70, were detected in the dataset, and 3 of the 18 proteins had a significant negative correlation with survival (Figure 4A-C). Of these, LOXL1 had the most significant correlation and was the only 1 of the 3 proteins that was correlated with survival at the mRNA level in an independent dataset (TCGA, GDC) (Figure 4D). LOXL1 is located in the endoplasmic reticulum membrane, therefore presenting a more difficult target, however, it is also secreted into the extracellular matrix, presenting an opportunity to therapeutically target the protein. Evidence from other studies shows that extracellular LOXL1 drives ECM remodeling and promotes cancer cell proliferation and invasion, further supporting its potential as a therapeutic target.^38^^,^^39^

Furthermore, when inputting the 18 common membrane proteins into the National Cancer Institute Bio Discovery Portal (Glioma-BioDP), the overexpression of mRNA coding for 18 genes was associated with a worse survival in the full patient cohort (Kaplan-Meier analysis, logrank *P*-value <.001). When separating the patient cohort into the 4 GBM subclasses, it was observed that overexpression of the 18 genes was associated with a significantly worse survival in the classical, mesenchymal, and proneural subclass, but not in the neural subclass (logrank *P*-value .004, .001, .004, and .166, respectively). Additionally, there was no significant change in survival when looking at the MGMT hazard ratio in each of these cohorts (data not shown). Whilst prognostic indices are not so informative of prognosis predictions for GBM (due to the median survival being so low), this data does suggest that some of these membrane proteins could exhibit a functional role in GBM survival and progression.

### Molecular Docking Identifies Candidate Therapeutics for Key Membrane Proteins

Membrane proteins ubiquitously overexpressed in GIN cells relative to non-neoplastic astrocytes make appealing candidate targets for future GBM therapies targeting residual disease in the invasive margin post-surgery. However, review of the literature revealed limited specific small molecule inhibitors targeting these compounds that could be repurposed for GBM therapy. Of the 18 commonly upregulated membrane proteins, a targeted, specific small-molecule inhibitor could only be identified for one protein. Myriocin was identified as an inhibitor of SPTLC and has been shown to reduce the proliferation of malignant melanoma cells.^40^

Alternative therapeutic modalities were identified for other candidate proteins. For example, there have been efforts to develop antibody-drug conjugates (ADCs) and immunotherapies against CD70 or CD58.^41^^,^^42^ Furthermore, Guo et al used virtual screening of 183,000 small molecule inhibitors followed by *in vitro* analysis to discover 5 potential CD58 small molecule inhibitors for colorectal cancer, with IC50 < 100 µM.^43^ Similarly, it may be possible to target SERPINE2 overexpression in GBM using inhibitors of upstream druggable targets. SERPINE2 expression may be upregulated by oncogenic activation of Ras, BRAF, and MEK1.^44^ Ras, specifically, is located on the plasma membrane and has many small molecule inhibitors against it.^45^

**Figure 5.**
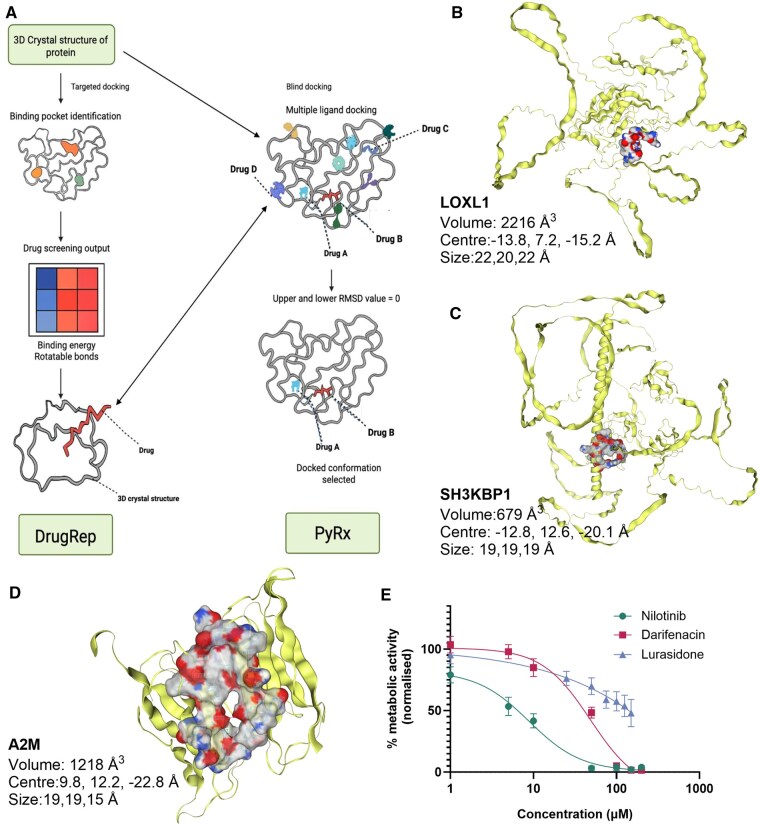
Molecular docking analysis leads to efficacious drug selection against glioblastoma invasive margin cell lines. (A) A schematic to show the molecular docking pipeline to identify repurposed drugs against proteins of interest. DrugRep was employed to identify binding pockets on each protein’s 3D crystal structure before identifying repurposed drugs with a high binding affinity to the pockets. PyRx was also utilized to carry out targeted docking of repurposed drugs to the precise location of the binding pocket identified in DrugRep on the 3D crystal structure, identifying those drugs with an RSMD value of 0, ie a perfect fit. These selected drugs were further filtered to identify the most promising candidates. The 3D crystal structures of each protein and their corresponding binding pockets are highlighted for (B) LOXL1, (C) SH3KBP1 and (D) A2M. Each protein is labeled with its corresponding binding pocket volume, where a drug could fit; its center which gives the coordinates (x, y, z) marking the exact point of the binding pocket; and size (length, width, height) of the grid box which outlines the region around the binding pocket that was explored during docking these coordinates. These were inputted into PyRx for targeted multiple ligand docking. Nilotinib, darifenacin and lurasidone were identified as the ideal candidates for these binding pockets. (E) Average dose response curves of each drug is shown against GIN8, GIN28 and GIN31 cell lines. Nilotinib and darifenacin were added at increasing concentrations from 1 μM to 200 μM. Lurasidone was added in increasing concentrations from 1 μM to 150 μM. Data was normalized to the carrier control and analyzed using the non-linear regression function. Data is shown as mean ± SEM after 72 h incubation.

To address the dearth of relevant small molecule inhibitors targeting the proteins of interest, a molecular docking approach was taken to identify compounds that could be repurposed as inhibitors of these proteins. DrugRep and PyRx were utilized to identify the top 10 approved drugs predicted to bind to each protein (Figure 5A). DrugRep encompasses an empirical scoring function called Cyscore that takes into consideration the curvature of the protein surface and therefore accounts for more nuanced protein-ligand interactions.

As demonstrated in Figure 5B-D, binding pockets with a volume ≥200 Å³ were subjected to screening against a library of FDA-approved drugs. The top 10 approved drugs were filtered based on their binding energy scores, with only those yielding a score of ≤−7 kcal/mol being advanced. The number of rotatable bonds within a structure is an important factor in dictating how flexible the ligand (drug) being docked will be. A range of 5-10 rotatable bonds was selected, as the “extended rule of five” states that 10 or fewer rotatable bonds would lead to good absorption and permeability of the drug, whilst too few rotatable bonds causes the drug to be too rigid, and too many rotatable bonds increases computational complexity, which can lead to less accurate outputs.^46^ Once passing the 2 criteria mentioned prior, the successful drug candidates underwent a second round of drug docking using PyRx, whereby RMSD value of 0 denotes a perfect conformational fit. The filtered drug list returned 55 candidates, which are listed in Supplementary Table S4. This combined approach enabled a comprehensive evaluation of drug-target interactions, ensuring only the most promising candidates were further advanced.

For these 55 candidates, literature searches were conducted to gather evidence of any cytotoxic effects. There was no existing literature of any cytotoxic effect of 10 of these candidates, thus, they were excluded. Out of the remaining 45 candidates, 10 had already been tested in clinical trials for GBM (leucovorin, folic acid, sulfasalazine, ibrutinib, cabozantinib, imatinib, regorafenib, sorafenib, pexidartinib, and irinotecan), therefore, it was deemed unnecessary to analyze them further. Candidates were then further filtered by the number of hydrogen bond donors (≤3) and acceptors (≤7), logP (1.0-4.6), and the molecular weight of the compounds (≤550 Da). Lipinski’s rule of five states that drugs should have 5 or fewer hydrogen bond donors and 10 or fewer hydrogen bond acceptors.^47^ Too many hydrogen bond donors or acceptors may impair a compound’s solubility and permeability, affecting the chances of crossing the BBB. The logP value indicates the lipophilicity of a compound and how soluble it will be. A low logP value allows the drug compound to be taken up by the body more readily due to the high-water content, but if this value is too low, the compound is unlikely to cross membranes. After filtering the candidate drugs based on these properties, 3 drugs were tested *in vitro* against the invasive margin cell lines, as displayed in Supplementary Table S5.

These drugs theoretically target LOXL1, SH3KBP1, and A2M, therefore, before undertaking drug screening, we sought to validate their presence using Western blot analysis. The use of E-Cadherin antibody demonstrated clear presence of this membrane protein in the membrane ­fraction, with a less dense band observed in the total protein fraction. Furthermore, each protein was present in GIN8, GIN28, and GIN31, corroborating LC-MS results (Supplementary Figure S5). SH3KBP1 had stronger bands in the total protein fraction, yet the active receptor-bound proteins are still present in the membrane protein fractions.

As displayed in Figure 5E, nilotinib (suggested to target LOXL1) and darifenacin (suggested to target SH3KBP1) produced average IC_50_ values of 8.45 µM and 46.77 µM, respectively. Previous work has already explored the functional relevance of both LOXL1 and SH3KBP1 in glioblastoma. Yu et al used gain- and loss-of-function approaches to show that LOXL1 promotes glioma cell survival through antiapoptotic activity, supporting the idea that LOXL1 inhibition may have therapeutic value.^48^ Similarly, Song and colleagues demonstrated that SH3KBP1 knockdown markedly reduced GBM cell proliferation, migration, and glioma stem cell self-renewal *in vitro*, as well as tumor growth in xenograft models. They further showed that SH3KBP1 directly regulates EGFR, a central driver of GBM progression, reinforcing its potential as a therapeutic target.^29^

Lurasidone did not reach an IC_50_ up to 150 µM; however, it has shown promise preclinically as a chemosensitizer, which could be tested further against GBM. Nevertheless, with 2 out of 3 compounds tested demonstrating efficacy against multiple patient-derived cell lines, these findings support the potential of this pipeline to nominate plasma-membrane-associated candidates for further validation, while not implying that the observed effects confirm on-target activity. Furthermore, to the best of our knowledge darifenacin has not been tested against GBM previously. Whilst Nilotinib has been tested against U251 spheroids,^49^ which are derived from the tumor core, the present work indicates that nilotinib is effective against invasive margin cells. Nevertheless, these repurposed drugs are known to act on additional cellular targets (nilotinib: BCR-ABL tyrosine kinase; darifenacin: M3 muscarinic acetylcholine receptor). As such, they may influence both their established targets and the proteins identified in our study. While it is encouraging that both compounds induce cytotoxicity in GIN cell lines, further validation of the specific targets will be necessary in future work.

Although these findings are encouraging, additional work is needed to validate the functional roles of these proteins within the invasive margin. Expanding the dataset to include cell lines or patient-derived tissue representing the full spectrum of GBM subtypes would help determine whether subtype-specific differences exist. Moreover, analyzing patient tissue (eg via patient-derived explant cultures) rather than cell lines cultured in FBS would mitigate concerns about culture-induced alterations. While comparisons with fetal HAs must be interpreted cautiously, the observation of GBM-associated proteins consistent with other reports suggests that astrocyte control retains some utility.

## Conclusion

This study identifies a consistent set of upregulated membrane proteins across three GBM invasive margin cell lines, highlighting their potential as broadly targetable vulnerabilities despite inter-patient heterogeneity. Quantitative proteomics revealed 18 significantly elevated membrane proteins (13 at the plasma membrane) with DYSF, LOXL1, and ROBO1 associated with poorer patient survival. Although many lack existing inhibitors, opportunities include targeting upstream regulators, repurposing small-molecule drugs, and developing antibody-drug conjugates. Docking studies yielded several promising candidates, two of which showed low-µM efficacy *in vitro*. Future work should validate these targets in heterogeneous patient samples using multiplex cytometry and assess functional relevance through drug-response and gene-knockdown studies.

## Supplementary Material

vdag181_Supplementary_Data

## Data Availability

The mass spectrometry proteomics data (acquisition files from the mass spectrometer and DIA-NN output files) have been deposited to the ProteomeXchange Consortium (http://proteomecentral.proteomexchange.org) via the PRIDE partner repository with the dataset identifier PXD058999.

## Cell Line Availability

All GBM primary cell lines reported in this study are available for sharing by contacting the corresponding author.
