# Supplementary material for: Membrane proteomic profiling to identify candidate therapy targets for glioblastoma infiltration
Source: Neurooncol Adv. 2026 Jul 10;8(1):vdag181. doi: 10.1093/noajnl/vdag181 (PMC13420503; doi:10.1093/noajnl/vdag181)
Supplement: vdag181_Supplementary_Data [file vdag181_supplementary_data.docx]

**Table S1. Glioma INvasive margin cell line patient information**

| **Cell** **Line** | **Age (Sex)** | **ATRX status** | **MGMT methylation (Y/N)** | **Resection** | **Treatment**  **(post-surgery)** | **Survival (post-surgery)** |
| --- | --- | --- | --- | --- | --- | --- |
| **GIN8** | 54 F | Intact | N | 92% | Gliadel wafers, 60 Gy radiotherapy, concurrent and adjuvant temozolomide | 5 months |
| **GIN28** | 71 M | Intact | N | 99% | N/A | 3 months |
| **GIN31** | 57 F | Intact | N | 100% | 60 Gy radiotherapy, concurrent and adjuvant temozolomide | 16.1 months |

**
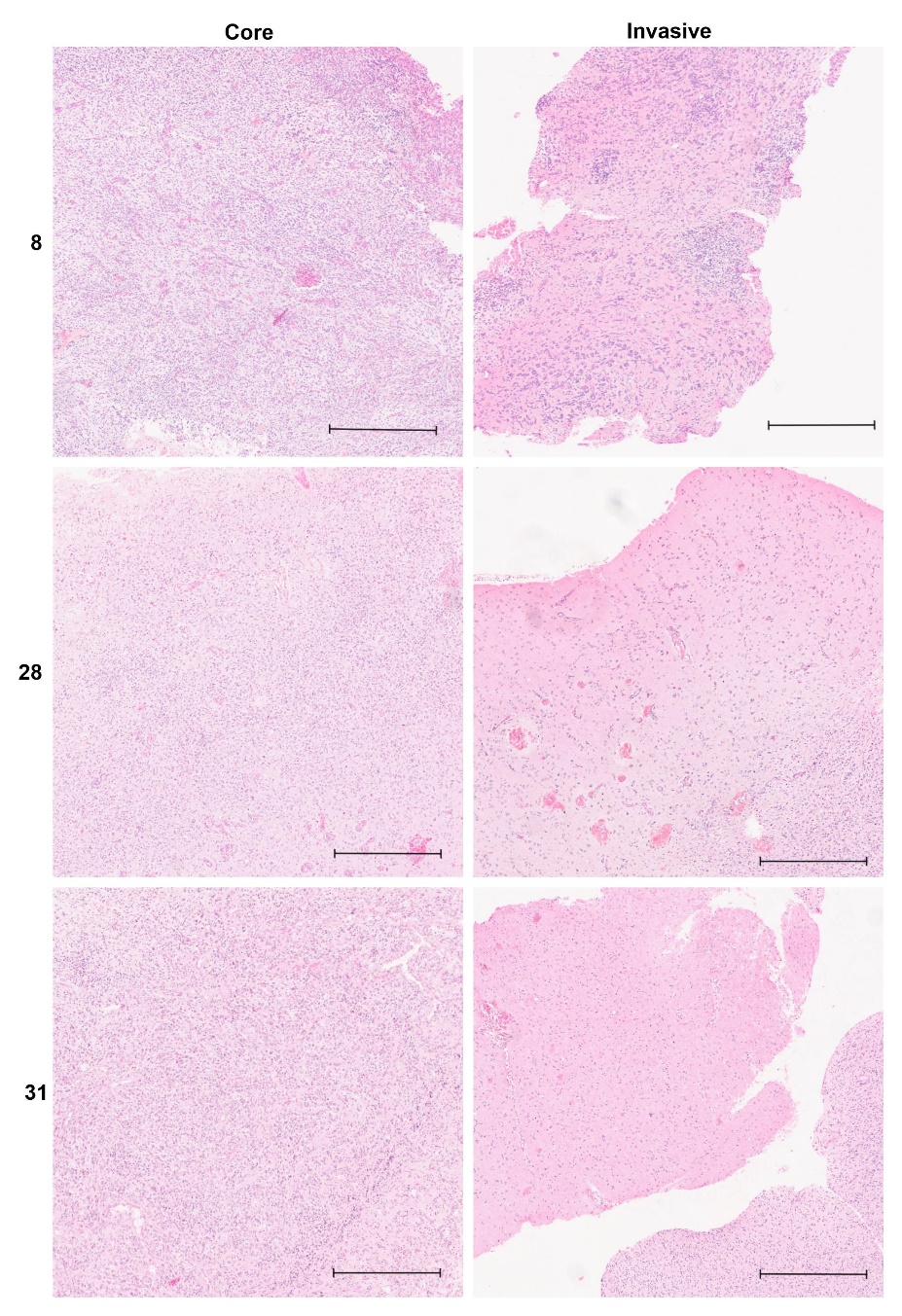
**

**Supplementary Figure 1. H&E stained sections of patient core and invasive tumour tissue, whereby each cell line was derived from the invasive region.** Patient 8, 28 and 31 correspond with GIN8, GIN28 and GIN31 respectively. Each image is at 5x magnification, scale bar = 500 µm.

**Table S2. Number of significant differentially abundant proteins before and after multiple hypothesis testing correction of *p* values.**

| Analysis (condition vs control) | Replicates | # Significant before p value correction | # Significant after p value correction |
| --- | --- | --- | --- |
| HA MP vs HA TP | 3 | 715 | 681 |
| GIN8 MP vs GIN8 TP | 3 | 1207 | 1195 |
| GIN28 MP vs GIN28 TP | 3 | 1068 | 1058 |
| GIN31 MP vs GIN31 TP | 3 | 924 | 912 |
| GIN8 TP vs HA TP | 3 | 366 | 333 |
| GIN8 MP vs HA MP | 3 | 280 | 229 |
| GIN28 TP vs HA TP | 3 | 308 | 276 |
| GIN28 MP vs HA MP | 3 | 262 | 212 |
| GIN31 TP vs HA TP | 3 | 378 | 360 |
| GIN31 MP vs HA MP | 3 | 321 | 267 |


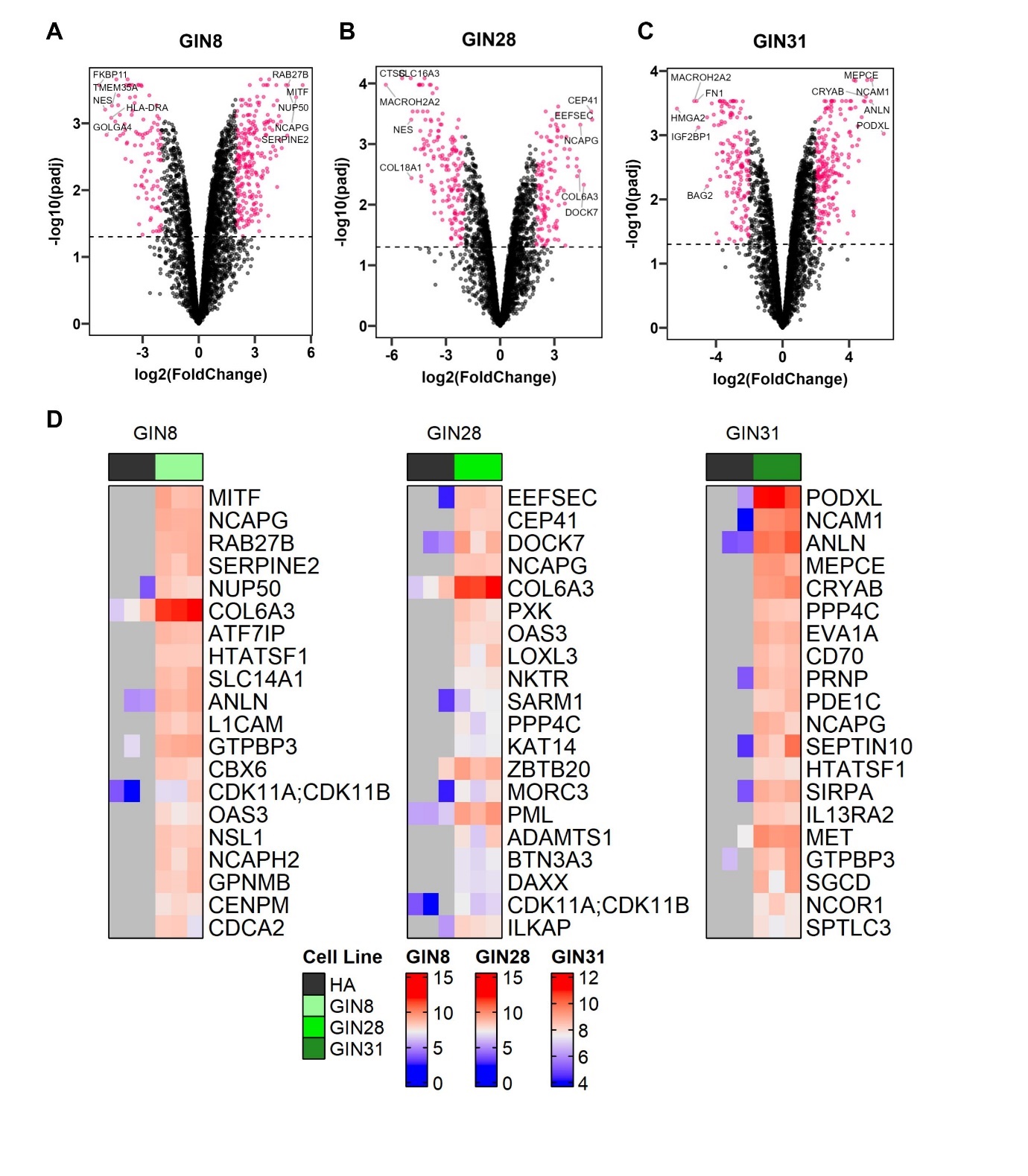


**Supplementary Figure 2: Analysis of GBM vs HA total protein fraction.** Volcano plots displaying (A) GIN8 232 Upregulated and 101 Downregulated proteins, (B) GIN28 115 Upregulated and 161 Downregulated proteins, and (C) GIN31 205 Upregulated and 155 Downregulated proteins. (D) Heatmaps showing the top 20 (highest log2FC) upregulated proteins from the total protein fraction GIN8, GIN28, and GIN31 cells compared to HA. Heatmap colour indicates log2 transformed protein expression. Grey indicates that protein was not detected in this sample.


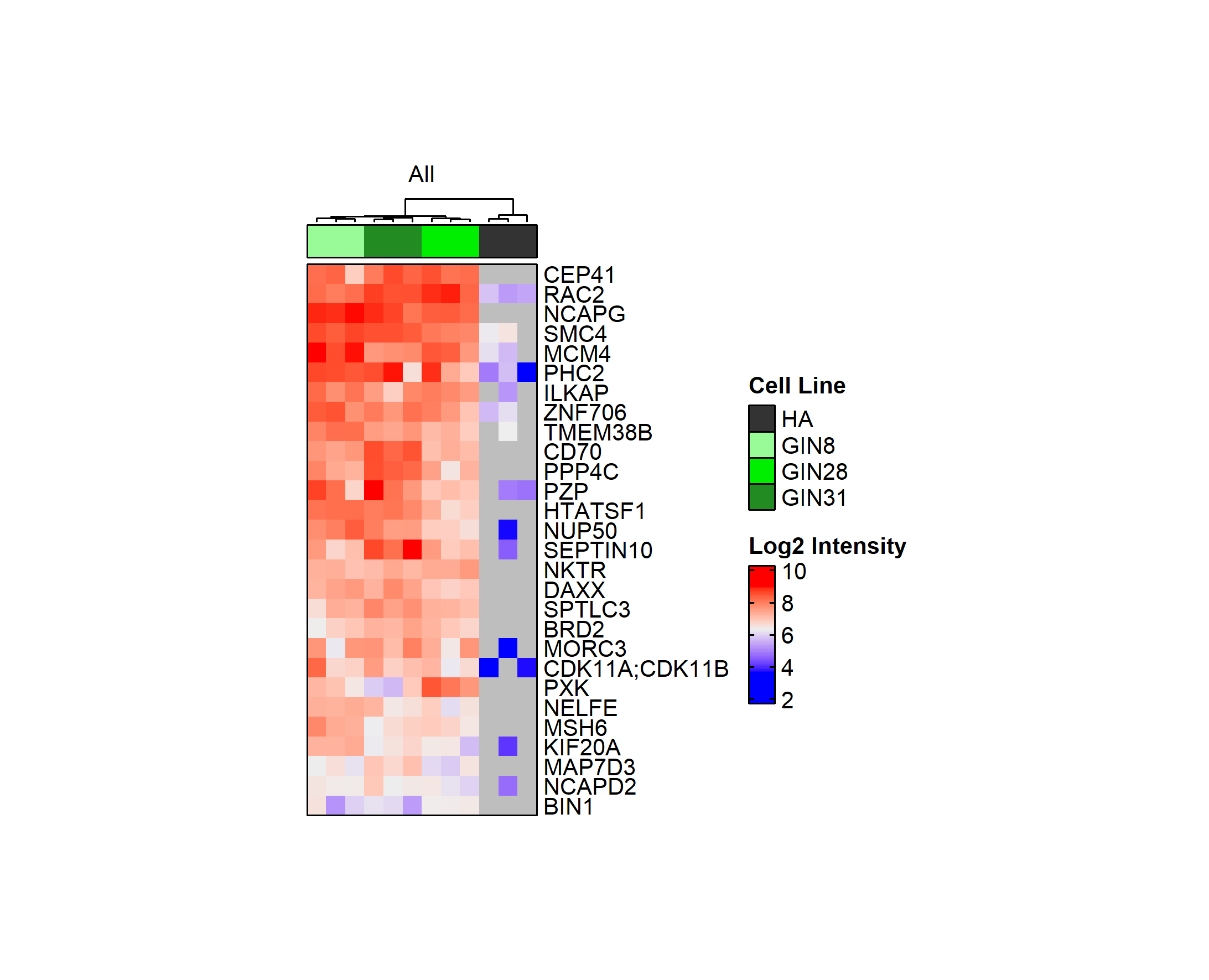


**Supplementary Figure 3: Commonly upregulated proteins in the total protein fraction among GIN cell lines.**​Heatmap showing the 28 proteins identified as being expressed at a higher level in all three GIN cell lines compared to HA. Colour represents the log2 transformed protein expression intensity from MS proteomics. Column dendrogram shows Euclidean clustering of samples. Grey indicates that the protein of interest was not detected in a particular sample.

**Table S3. Common total proteins upregulated across all 3 GIN lines when compared to HA.** (absolute log2 fold change > 2, *p*-adj value < 0.05)

| Protein | | Function | Protein | | Function |
| --- | --- | --- | --- | --- | --- |
| 1 | **BIN1** | Differentiation, endocytosis | **15** | **NCAPD2** | Cell division |
| 2 | **HTATSF1** | DNA repair | **16** | **PXK** | Actin binding |
| 3 | **KIF20A** | Protein transport | **17** | **MAP7D3** | Microtubule formation |
| 4 | **RAC2** | Signaling protein | **18** | **PHC2** | Developmental protein |
| 5 | **NELFE** | Transcription regulator | **19** | **NCAPG** | Cell division |
| 6 | **PZP** | Immune regulation | **20** | **CEP41** | Protein transport |
| 7 | **CDK11A;CDK11B** | Cell cycle regulator | **21** | **ILKAP** | Protein phosphatase |
| 8 | **BRD2** | Chromatin regulator | **22** | **SMC4** | Cell division |
| 9 | **NKTR** | Isomerase | **23** | **SPTLC3** | Lipid metabolism |
| 10 | **CD70** | Immune signalling | **24** | **TMEM38B** | Ion transport |
| 11 | **MCM4** | DNA replication | **25** | **SEPTIN10** | Cell division |
| 12 | **MSH6** | DNA repair | **26** | **DAXX** | Chromatin regulator |
| 13 | **PPP4C** | DNA repair | **27** | **NUP50** | Nuclear protein import |
| 14 | MORC3 | Innate immunity | 28 | ZNF706 | Translation regulation |


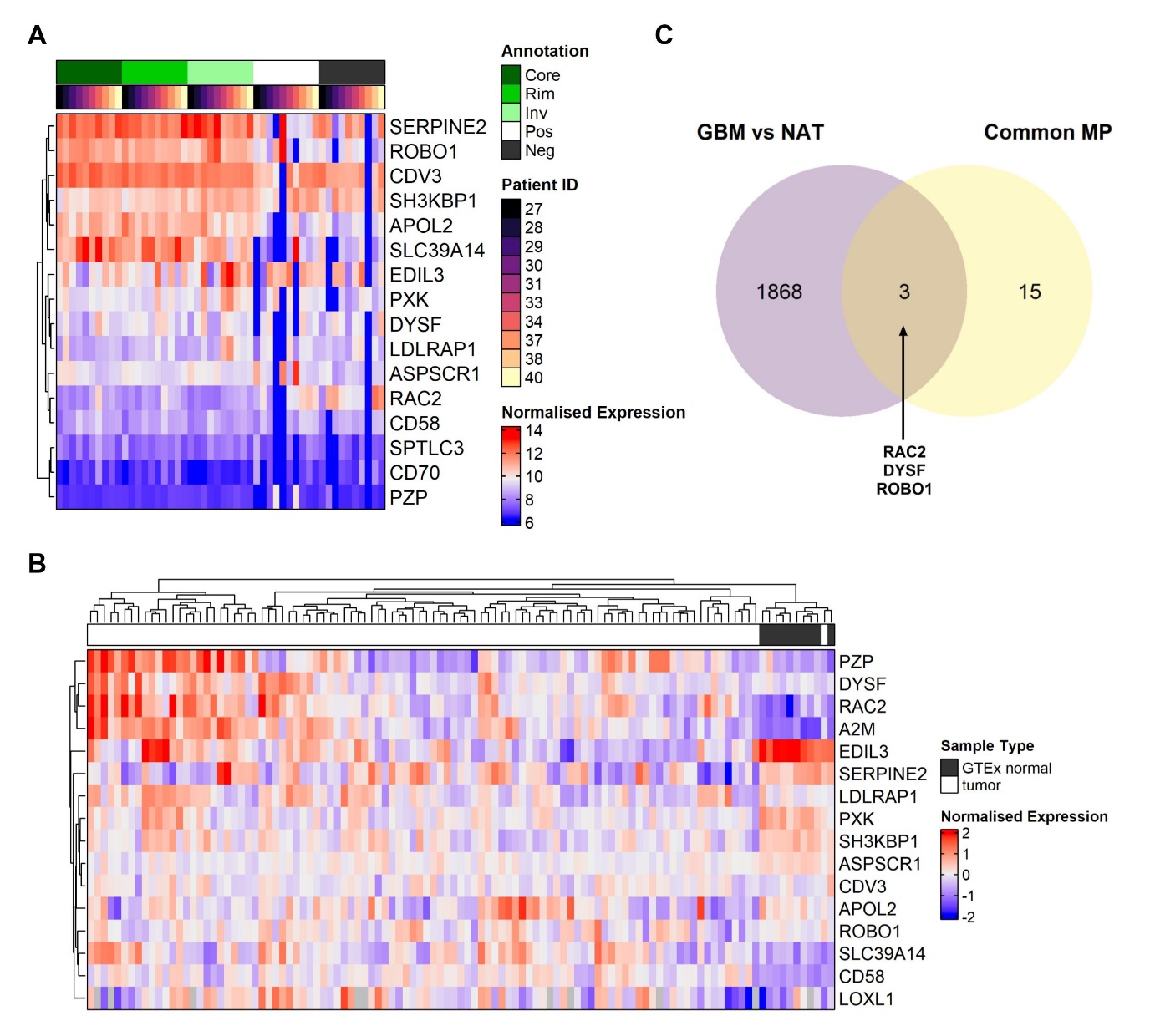


**Supplementary Figure 4: Comparison to other studies which have analysed normal brain tissue**. (A) ​ VST normalised gene counts from RNA-sequencing previously conducted on GBM intra-tumour regions including for patients 28 and 31 for which we also generated GIN cell lines GIN28 and GIN31. Confirming that mRNA expression of 16 of the 18 commonly identified GIN membrane proteins occurs in GBM tissues.  (B) Heatmap showing Log2 transformed, normalised, and batch corrected global proteomics data from REFERENCE composed of 99 GBMs and 10 unmatched healthy brain tissue controls.​(C) ​ Venn diagram comparing proteins identified as being more abundant in all three GIN cell lines relative to HA, and those reported as being significantly more abundant in primary GBM tissue relative to normal adjacent tissue (NAT) in Tatari et al.

​

**Table S4. Potential drug candidates remaining following PyRx molecular docking**, prior to additional filtration steps.

| Proteins | Drugs remaining after PyRx docking |
| --- | --- |
| DYSF | Digoxin, acetyldigitoxin |
| A2M | Leucovorin, levoleucovorin, elexacaftor, lurasidone, sulfasalazine, folic acid |
| SERPINE2 | Acetyldigitoxin, chlorhexidine, ergocalciferol, ibrutinib, digitoxin |
| PZP | Acetyldigitoxin, digitoxin, cabozantinib, imatinib, naldemedine |
| CD70 | Tucatinib, delamanid, eltrombopag, troglitazone |
| LOXL1 | Glecaprevir, entrectinib, nilotinib, dihydroergocristine |
| SLC39A14 | Regorafenib, tucatinib, novobiocin, delamanid, pimozide, sorafenib |
| SH3KBP1 | Regorafenib, tucatinib, eltrombopag, pimozide, zafirlukast, sorafenib, darifenacin, lumacaftor |
| APOL2 | Aripiprazole, mirabegron, pexidartinib |
| ASPSCR1 | Dexamethasone metasulfobenzoate, regorafenib, sorafenib, pexidartinib, alatrofloxacin, lymecycline |
| SPTLC3 | Raltegravir, lumacaftor, cromoglicic acid, glimepiride, irinotecan, fluspirilene |

**Table S5. Final drug candidates tested on glioblastoma invasive margin cells after molecular docking and filtering**

| Name | Target | Previous use(s) |
| --- | --- | --- |
| Nilotinib | LOXL1 | Chemotherapeutic, CML^48^ |
| Darifenacin | SH3KBP1 | Muscarinic receptor antagonist (gastric^49^ and prostate^50^ cancer efficacy pre-clinically) |
| Lurasidone | A2M | Antipsychotic (chemo-sensitiser pre-clinically^51^) |


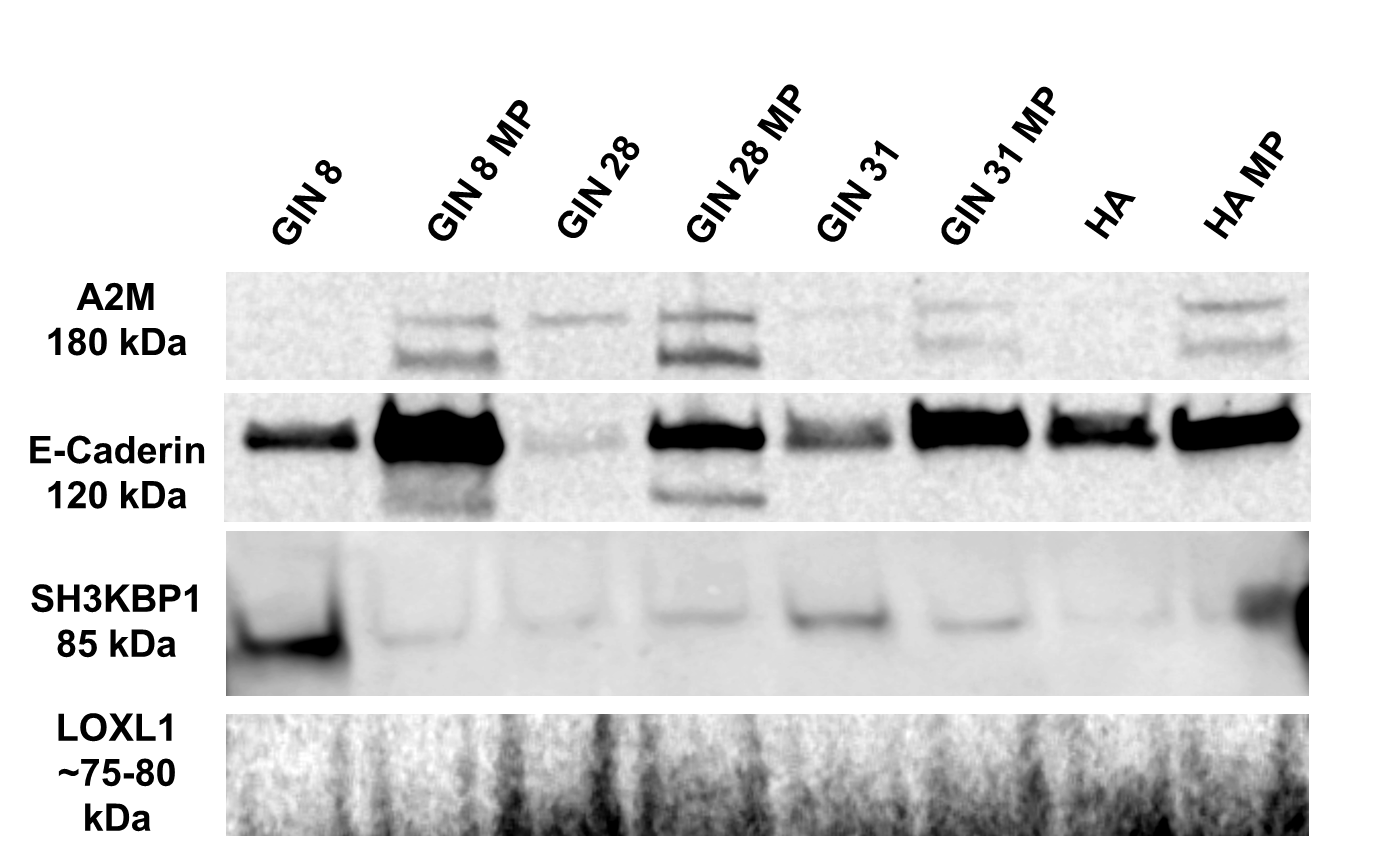


**Supplementary Figure 5. Western Blot analysis of E-Cadherin, A2M, LOXL1 and SH3KBP1 in GIN8. GIN28, GIN31 and HA.** Both total and membrane protein fractions were analysed. Presence of A2M and SH3KBP1 is indicated in all cell lines, with denser bands observed in GIN8 and GIN28. LOXL1 presence is also indicated, although the bands are not clear enough to allow for comparison between cell lines. Finally, a more intense E‑cadherin band is detected in the membrane fraction of all cell lines when compared with the corresponding total protein fraction.
